# Modeling the Effects of Formulary Exclusions: How Many Patients Could Be Affected by a Specific Exclusion?

**DOI:** 10.36469/001c.94544

**Published:** 2024-03-25

**Authors:** Anne M. Sydor, Emily Bergin, Jonathan Kay, Erik Stone, Robert Popovian

**Affiliations:** 1 1. Division of Patient-Focused Economic and Policy Research Global Healthy Living Foundation, Upper Nyack, New York, USA; 2 Division of Patient-Focused Economic and Policy Research Global Healthy Living Foundation, Upper Nyack, New York, USA; 3 Pioneer Institute, Boston, Massachusetts, USA; 4 Progressive Policy Institute, Washington, DC, USA

**Keywords:** formulary exclusion, formularies, pharmacy benefits management, prescription drug policy, patient-centered care

## Abstract

**Background:** Medication formularies, initially designed to promote the use of cost-effective generic drugs, are now designed to maximize financial benefits for the pharmacy benefit management companies that negotiate purchase prices. In the second-largest pharmacy benefit management formulary that is publicly available, 55% of mandated substitutions are not for generic or biosimilar versions of the same active ingredient and/or formulation and may not be medically or financially beneficial to patients.

**Methods:** We modeled the effect of excluding novel agents for atrial fibrillation/venous thromboembolism, migraine prevention, and psoriasis, which all would require substitution with a different active ingredient. Using population data, market share of the 2 largest US formularies, and 2021 prescription data, we calculated how many people could be affected by such exclusions. Using data from the published literature, we calculated how many of those individuals are likely to discontinue treatment and/or have adverse events due to a formulary exclusion.

**Results:** The number of people likely to have adverse events due to the exclusion could be as high as 1 million for atrial fibrillation/venous thromboembolism, 900 000 for migraine prevention, and 500 000 for psoriasis. The numbers likely to discontinue treatment for their condition are as high as 924 000 for atrial fibrillation/venous thromboembolism, 646 000 for migraine, and 138 000 for psoriasis.

**Conclusion:** Substitution with a nonequivalent treatment is common in formularies currently in use and is not without substantial consequences for hundreds of thousands of patients. Forced medication substitution results in costly increases in morbidity and mortality and should be part of the cost-benefit analysis of any formulary exclusion.

## BACKGROUND

The US has the highest expenditures and the worst health outcomes among high-income countries.^1^ Efforts to reduce costs often focus on the costs of prescription medicines, even though biopharmaceutical spending accounts for less than 9% of healthcare expenditures^2^ and grew less rapidly than other healthcare costs from 2010 to 2019.^3^ When considering biopharmaceutical pricing in the United States (US), it is essential to understand that insurers do not pay the medication list price. Instead, insurers pay a lower price negotiated by pharmacy benefit management (PBM) companies. There is now increased scrutiny on PBMs with an ongoing US Federal Trade Commission (FTC) investigation^4^ and a proposal to regulate PBMs in Congress.^5^ This scrutiny is related to evidence that the discounts PBMs negotiate are not passed on to patients^6^ and may artificially increase medication list prices.^7^ We have also shown, in prior analyses,^8,9^ that PBM formularies often exclude specific medicines in ways that may be harmful to individual patients. Our prior analyses assessed the proportion of existing exclusions in one national, publicly available PBM formulary that were not necessarily beneficial to an individual patient. In the current analysis, we evaluate *how many individuals might be adversely affected* if a currently covered medication were to be excluded in either of the 2 largest US PBM formularies (Express Scripts International [ESI] and CVS Health). Because hundreds of medications are subject to inclusion or exclusion each year, we limited this analysis to agents that are approved for the treatment of atrial fibrillation (AFib)/venous thromboembolism (VTE), migraine, and psoriasis.

### The Impact of PBM Formulary Practices

Formularies are a PBM tool originally intended to promote the use of cost-effective treatment, prioritizing less costly treatments.^10^ In effect, formularies increased the utilization of generic equivalent drugs.^11^ A generic equivalent is a medication with the same active ingredient and formulation as the patented brand-name medication, intended to have the same clinical effect for the patient as the brand-name medication.^12^

Today, formularies are constructed to limit the number of medicines available for patients based on financial gains for the PBMs,^13^ and many mandated exclusions or substitutions are not equivalent.^14^ Also, it is impossible to determine whether formulary changes are less costly for patients, employers, or the government because the list prices of biopharmaceuticals are not the price that a PBM or an insurer pays. Instead, insurers and PBMs negotiate steep, nontransparent concessions, lowering the net price for only the PBMs and insurers. Unfortunately for patients, the list price determines their out-of-pocket costs related to co-insurance and deductibles.^15^ Less than 1% of the concessions received by PBMs and insurers are passed on to the patients.^6^

From a therapeutic standpoint, the substitutions and exclusions often are not medically beneficial or appropriate for most patients. Our previous research, published in 2022,^8^ demonstrated that almost half of all exclusions mandated by the second largest PBM in the US marketplace, ESI, were nonequivalent. Such exclusions force patients to use medications with a different active ingredient, formulation, or mode of administration than their clinician had prescribed.^8^ In some cases, treatments were excluded with no alternative, effectively denying patients any treatment. In our updated 2023 research, the proportion of exclusions by ESI with questionable medical and economic benefits to patients increased to more than half (57.4%).^9^

### Number of People in the US with Commercial and Medicare Part D Pharmacy Benefits Provided through PBMs

**Figure 1.**
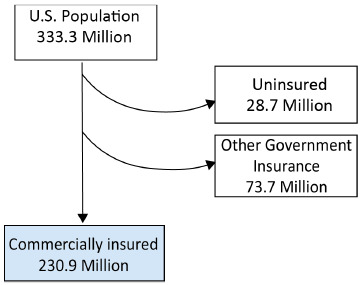
Number of People Affected by Commercial and Medicare Part D Formularies in the US The 2022 market share for the 2 largest publicly available formularies, CVS Caremark Performance Standard Control (CVS) and Express Scripts National Preferred (ESI), is 33% and 24%, respectively.^19^

In 2022, the US Census Bureau estimated the US population as 333.3 million individuals.^16^ Data from the Kaiser Family Foundation for 2021 showed that 48.5% of the US population (~161.6 million people) had private insurance through their employer, 6.1% (~20.3 million people) had small group private insurance, and 8.6% (~28.7 million people) had no health insurance.^16,17^ Another Kaiser Family Foundation report showed that 14.7% of the US population (~48.9 million) had Medicare Part D coverage.^18^ Taken together, these numbers suggest that 69.3% of the US population (~230.9 million people) were covered by commercial or Medicare Part D formularies, with the remaining 22.1% of the US population (~73.7 million) covered by other government insurance (**Figure 1**).

### Medication Classes Evaluated

We evaluated medication classes currently affected by CVS Health and ESI formulary exclusions and utilized to treat cardiovascular, neurologic, and dermatologic conditions. We chose these classes since they all require therapeutic substitution due to the formulary exclusion policy. Therapeutic substitution occurs when an insurer or PBM mandates a patient to utilize a medicine that does not possess the same active ingredient as what was prescribed by their provider. Exclusion of treatments in all three medication classes forces discontinuation or substitution of therapy, leading to adverse patient outcomes.

## METHODS

### Data Sources

**Formulary data:** We used the CVS Health and ESI national formularies because both organizations publish a transparent national formulary and identify their excluded medicines.

**Literature search parameters**: We searched the published medical literature and the gray web to obtain data for our calculations and modeling of patient outcomes. We used the PubMed.gov and EMBASE databases to search published peer-reviewed medical literature. We searched the gray web with the Google search engine. For the prevalence of each indication evaluated, we used that indication(s) joined with the operator “AND” to “prevalence OR epidemiology” as search terms. For the proportion of people likely to discontinue treatment or have a related adverse event (the medical consequence of discontinuation or side effect of a new medicine) after an exclusion, we used the search terms “nonmedical switching OR formulary exclusion” either alone or joined by “AND” to the medication classes (anticoagulants or tumor necrosis factor inhibitors) or diseases (migraine, Afib/VTE, and psoriasis) being evaluated. In the PubMed and EMBASE databases, we limited search terms for indications, prevalence, and epidemiology to Medical Subject Hypertext (MESH) terms or Emtree subjects. We limited all searches to English-language articles published from 2002 to 2022 and completed all queries on March 31, 2023. We reviewed the publication type and titles of all publications returned by the search queries. When fewer than 10 articles were available for inclusion, we included the references cited in those articles in our search for relevant published data.

*Inclusion and exclusion criteria*: For data on the prevalence of the indications being studied, only epidemiological studies that included all subtypes of the indication (rather than the indication in a specific population) and included data from the US were included. We excluded review articles, letters, and studies that did not include data from the US or were about specific subgroups with the indication of interest; for example, only epidemiologic studies of all people with atrial fibrillation were included, whereas studies of atrial fibrillation in a subset having a specific procedure, on dialysis, or with heart failure were excluded. We excluded papers without abstracts. We reviewed the abstracts and text for all remaining articles and then applied the same inclusion and exclusion criteria. Additionally, we included only articles that provided US-specific prevalence estimates based on cross-sectional, multicenter studies or analysis of large national databases. We derived prevalence ranges by identifying the low and high estimates across at least 3 articles with overlapping ranges.

For data on the effects of nonmedical switching, we excluded review articles, letters, and editorials. We reviewed all remaining abstracts and excluded articles that reported data only from patients who voluntarily switched treatments as part of participating in a study. We reviewed the full text of the remaining articles and excluded articles that did not report discontinuation rates after an involuntary nonmedical switch or did not report adverse events. We included articles that reported data on discontinuation of treatment after an involuntary, nonmedical switching or on adverse events that occurred after discontinuing or switching medications for any reason.

*Medication market share data*: We obtained market share data for the medications evaluated from IQVIA, a private company that owns proprietary databases that are utilized by many entities in the evaluation of the biopharmaceutical marketplace in the US. Within each class or subclass evaluated, we calculated the market share for each medicine as the total number of prescriptions for that medication divided by all prescriptions for all medications in the class or subclass evaluated.

### Calculating the Potential Effects of a Specific Exclusion

**Figure 2.**
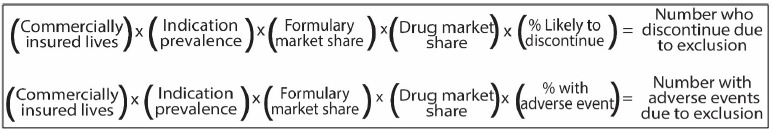
Calculation for the Number of Patients Affected by Specific Exclusion in a Specific Formulary

We calculated the number of people who could be affected by an exclusion as the number of individuals in the US with commercial or Medicare Part D insurance (commercially insured lives) multiplied by the indication prevalence, then formulary market share, and then medication market share (**Figure 2**; see **Supplementary Table** for list of data sources for each variable).^19^ We then calculated the number of people likely to discontinue therapy or have an adverse outcome due to an exclusion using our findings from the literature on discontinuation rates and adverse events (disease worsening from discontinuation plus adverse effects of switching to a new biopharmaceutical treatment).

## RESULTS

### Disease Prevalence for Indications Evaluated

**Figure 3.**
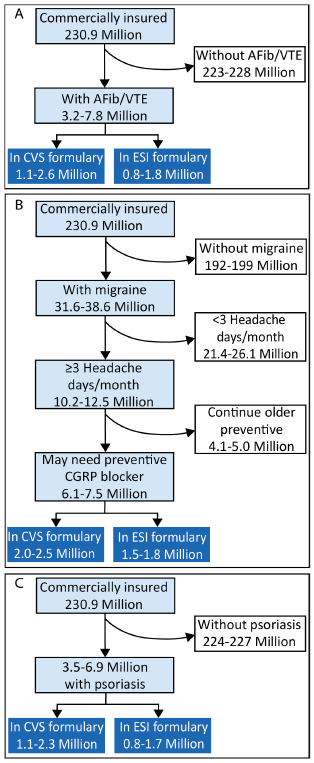
Number of People Potentially Affected by Exclusion of a Brand-Name Anticoagulant (**A**), Migraine Preventive (**B**), or Antipsoriatic (**C**) Medication Abbreviations: AFib/VTE, atrial fibrillation/venous thromboembolism; CGRP, calcitonin gene-related peptide; ESI, Express Scripts International.

*Anticoagulants* are indicated for the treatment of atrial fibrillation (AFib) and/or venous thromboembolism (VTE), including pulmonary embolism and deep vein thrombosis. Epidemiologic studies show the age-adjusted prevalence of AFib in the US is 1% to 2.9%.^20–22^ Nominal data are available on the prevalence of VTE, with a 2011 report of 0.4% to 0.5% in the US.^23^ Based on these data and the market share of the CVS and ESI formularies,^19^ 800 000 to 2.6 million individuals could be affected by the exclusion of an anticoagulant by CVS or ESI (**Figure 3A**).

*Migraine preventive treatments* are indicated for people with migraine who have more than 3 headache days per month.^24^ The age-adjusted prevalence of migraine is 9.5% to 11.6% (~31.6 million–38.6 million cases). Among people with migraine, 33.3% have more than 3 migraine attacks per month, making them eligible for preventive treatment.^25–27^ However, many eligible patients do not receive preventive treatment. Among those who do, only 40% continue the use of older medicines that do not block the activity of calcitonin gene-related peptide (CGRP) after 6 months.^28,29^ Based on these data and the market share of the CVS and ESI formularies, 1.5 million to 2.5 million individuals could be affected by excluding a migraine prevention treatment (**Figure 3B**) that blocks CGRP.

*Antipsoriatic agents* are indicated for people with topical psoriasis, for which prevalence in the US has consistently been reported as 1.5% to 3% (~3.5 million–6.9 million people).^30–32^ Based on these data and the market share of the CVS and ESI formularies,^19^ 800 000 to 2.3 million individuals could be affected by the exclusion of an antipsoriatic medication by CVS or ESI (**Figure 3C**).

### Rates of Discontinuation and Adverse Events After Switching

*Anticoagulants.* Studies show that 17% to 30% of patients discontinued all anticoagulant treatment upon exclusion of apixaban from a national formulary.^33,34^ Using this discontinuation rate, up to 389 000 and 535 000 patients, respectively, would likely discontinue treatment with any anticoagulant if excluded by ESI or CVS. For those who discontinue anticoagulation therapy, the likelihood of stroke or other cardiovascular events increases by 45% to 85%.^35^ In addition, 2% to 10% of patients who switched from anticoagulant medicines had an adverse event.^36–39^ We added the number of people likely to have a serious cardiovascular event (45%-85% of the 17%-30% who discontinue) to the number of people likely to have an adverse event after switching anticoagulants (2%-10% of the 83% who switch). This calculation shows that up to 422 000 and 580 000 patients may have an adverse event upon forced switching due to CVS Health and ESI formulary exclusions, respectively (**Table 1**).

**Table 1.** Effects of Excluding a Brand-Name Anticoagulant Medication

**Medication**	**Market Share, %**	**Potentially Affected, n**	**Likely to Discontinue, n**	**Adverse Events, n**
**Potential Effects of CVS Excluding a Brand-Name Anticoagulant (000s)**				
Eliquis (apixaban)	68.88	734–1784	125-535	68-580
Xarelto (rivaroxaban)	29.76	317-771	54-231	30-251
Pradaxa (dabigatran)	1.31	14-34	2.3-10	1.3-11
Savaysa (edoxaban)	0.06	0.6-1.5	0.1-0.5	0.06-0.5
**Potential Effects of ESI Excluding a Brand-Name Anticoagulant (000s)**				
Eliquis (apixaban)	68.88	534-1298	91-389	50-422
Xarelto (rivaroxaban)	29.76	231-561	39-168	21-182
Pradaxa (dabigatran)	1.31	10-25	1.7-7.4	0.9-8.0
Savaysa (edoxaban)	0.06	0.4-1.1	0.08-0.3	0.04-0.4

Abbreviation: ESI, Express Scripts International.

*Migraine preventive agents.* No published data were available on rates of discontinuing or switching CGRP-blocking medicines after involuntary, nonmedical switching. However, a meta-analysis of forced nonequivalent substitutions across multiple classes of medicines showed 9% to 19% of patients discontinue treatment.^40^ Based on these data, we calculated that a maximum of 374 000 individuals are likely to discontinue therapy due to a formulary exclusion by CVS Health or ESI. Regarding adverse events related to discontinuation, a published study documented increased migraine attack frequency after stopping a monoclonal antibody CGRP blocker for any reason.^41^ Another study showed stopping or switching any migraine preventive treatment increased migraine attack frequency and/or serious adverse events in at least half of patients.^42^ Based on these data, we calculated that 50% of patients, up to 986 000 people, would have worsening disease and other adverse events after the exclusion of a migraine preventive agent by ESI or CVS (**Table 2**).

**Table 2.** Effects of Excluding a Brand-Name Preventive Migraine Medication

**Medication**	**Market Share, %**	**Potentially Affected, n**	**Likely to Discontinue, n**	**Adverse Events, n**
**Potential Effects of CVS Excluding a Brand-Name Migraine Preventive Treatment (000s)**				
MAbs				
Emgality (galcanezumab)	42.56	858-1050	77-200	429-525
Aimovig (erenumab)	36.91	745-911	67-173	373-455
Ajovy (fremenezumab)	20.49	413-506	37-96	207-253
Vyepti (eptinezumab)	0.04	0.8-1	0.07-0.2	0.4-0.5
Gepants				
Nurtec (rimegepant)	79.89	1,612-1,971	145-374	806-986
Qulipta (ubrogepant)	20.11	406-496	37-94	203-248
**Potential Effects of ESI Excluding a Brand-Name Migraine Preventive Treatment (000s)**				
MAbs				
Emgality (galcanezumab)	42.56	625-764	56-145	312-382
Aimovig (erenumab)	36.91	542-662	49-103	271-331
Ajovy (fremenezumab)	20.49	301-368	27-70	150-184
Vyepti (eptinezumab)	0.04	0.6-0.7	0.05-0.14	0.3-0.4
Gepants				
Nurtec (rimegepant)	79.89	1172-1433	106-272	586-717
Qulipta (ubrogepant)	20.11	295-361	27-69	148-180

Abbreviations: ESI, Express Scripts International; MAbs, monoclonal antibody calcitonin gene-related peptide blocker.

*Antipsoriatic agents.* A meta-analysis showed that 6% to 9% of patients discontinue therapy upon being forced to switch tumor necrosis factor inhibitors.^43^ For other antipsoriatic medications, we used the 9% to 19% discontinuation rate seen in a meta-analysis of nonmedical switching across multiple treatment classes^40^ and in studies of nonmedical switching of biologics.^44^ The rate of adverse events from all of these agents is high, and studies show that switching results in 15% to 35% of patients having adverse events.^45–48^ Using these data, we calculate up to 28 000 patients may discontinue therapy, and up to 311 000 may have an adverse event because of a formulary exclusion (**Table 3**).

**Table 3.** Effects of Excluding a Brand-Name Antipsoriatic Medication

**Medication**	**Market Share, %**	**Potentially Affected, n**	**Likely to Discontinue, n**	**Adverse Events, n**
**Potential Effects of CVS Excluding a Brand-Name or Biosimilar Psoriasis Treatment (000s)**				
TNF inhibitors				
Humira (adalimumab)	38.92	445-890	27-80	67-311
Enbrel (etanercept)	13.18	151-301	9-27	23-105
Cimzia (certolizumab)	2.50	29-57	2-5	4-20
Simponi (golimumab)	1.41	16-32	1-3	2-11
Remicade (infliximab)	0.52	6-12	0.4-1	0.9-4
Inflectra (infliximab)	0.30	3-7	0.2-0.6	0.2-2
Renflexis (infliximab)	0.03	0.3-0.7	0.02-0.06	0.05-0.2
Avsola (infliximab)	0.02	0.2-0.5	0.01-0.04	0.03-0.1
Infliximab (infliximab)	0.02	0.2-0.5	0.01-0.04	0.03-0.1
Other				
Otezla (apremilast)	8.55	98-195	9-19	15-68
Cosentyx (ustekinumab)	7.30	83-167	8-32	13-58
Stelara (ustekinumab)	6.38	73-146	7-28	11-51
Taltz (ixekizumab)	6.17	71-141	6-27	11-49
Xeljanz (tofacitinib)	5.24	60-120	5-23	9-42
Tremfya (guselkumab)	3.46	40-79	4-15	6-28
Rinvoq (upadacitinib)	3.23	37-74	3-14	6-26
Skyrizi (risankizumab)	2.63	30-60	3-11	5-21
Siliq (brodalumab)	0.07	0.8-1.6	0.07-0.3	0.1-0.6
Ilumya (tildrakizumab)	0.06	0.7-1.4	0.06-0.3	0.1-0.5
**Potential Effects of ESI Excluding a Brand-Name or Biosimilar Psoriasis Treatment (000s)**				
TNF inhibitors				
Humira (adalimumab)	38.92	323-647	19-58	113-226
Enbrel (etanercept)	13.18	110-219	6.6-20	38-77
Cimzia (certolizumab)	2.50	21-42	1.2-3.7	7.3-15
Simponi (golimumab)	1.41	12-23	0.7-2.1	4.1-8.2
Remicade (infliximab)	0.52	4.3-8.6	0.3-0.8	1.5-3.0
Inflectra (infliximab)	0.30	2.5-5.0	0.2-.04	0.9-1.7
Renflexis (infliximab)	0.03	0.2-0.5	0.02-0.05	0.09-0.2
Avsola (infliximab)	0.02	0.2-0.3	0.01-0.03	0.06-0.1
Infliximab (infliximab)	0.02	0.2-0.3	0.01-0.03	0.06-0.1
Other				
Otezla (apremilast)	8.55	71-142	6.4-27	25-50
Cosentyx (ustekinumab)	7.30	61-121	5.5-23	21-42
Stelara (ustekinumab)	6.38	53-106	4.8-20	19-37
Taltz (ixekizumab)	6.17	51-103	4.6-19	18-36
Xeljanz (tofacitinib)	5.24	44-87	3.9-17	15-30
Tremfya (guselkumab)	3.46	29-58	2.6-11	10-20
Rinvoq (upadacitinib)	3.23	27-54	2.4-10	9.4-19
Skyrizi (risankizumab)	2.63	22-44	2.0-8.3	7.7-15
Siliq (brodalumab)	0.07	0.6-1.2	0.05-0.2	0.2-0.4

Abbreviation: ESI, Express Scripts International.

## DISCUSSION

This is the first study to model how many people could be affected by a potential exclusion of specific medications from national-level formularies in the US. Strengths of the study include the use of real-world data for the market share of a treatment and the number of people covered by commercial formularies. The data used to generate prevalence, discontinuation, and adverse event rates were derived from a systematic literature review and used to model low-to-high ranges for the number of people who could be affected. We used the low-to-high ranges to account for the variability that is inherent to estimates of prevalence, discontinuation, and adverse events. Data from this study adds to our body of knowledge regarding the scope and size effects of formulary exclusions by showing that for medications in 3 therapeutic classes with more than a 10% market share, hundreds of thousands to millions of people could discontinue treatment or experience adverse events and disease worsening. The model developed allows estimation of the number of people who would be affected for regions other than the US and other medication classes, as long as the prevalence of the indication is known. The model also enables evaluation of how many people would be affected by the exclusion of a specific biopharmaceutical as long as the prevalence of disease and market share for specific agents are available.

The uncertainty of disease prevalence and rates of discontinuation and adverse events contributed to the large ranges between low and high estimates; however, the largest source of variance was the market share. Notably, across all 3 therapeutic classes evaluated, any with more than a 5% market share could affect tens of thousands to millions of people. For example, excluding the most commonly used anticoagulant could increase the number of strokes by 227 000 to 459 000 within 6 months. Excluding the most frequently used new migraine preventive treatment would likely worsen the frequency of migraine attacks and increase disability for 741 000 to 904 000 patients. Excluding the most-used antipsoriatic medication could cause serious side effects for 180 000 to 500 000 patients.

Insurers and PBMs have argued that formularies promote cost-effective biopharmaceutical use and positive therapeutic outcomes, which was the original intent of developing formularies. However, formulary practices today evaluate cost-effectiveness based on the net price a BPM negotiates with the biopharmaceutical company, not the list price of the biopharmaceutical or patients’ share of pharmaceutical costs. Including a medication in a formulary provides PBMs leverage to negotiate higher rebates and discounts from the manufacturer. Such practices may explain why some formularies even prefer some brand-name medicines over generic drugs and biosimilars. Still, it is impossible to know this without knowing the actual net price paid for a medicine by the PBM or insurer. In fact, over half of the exclusions in the second-largest PBM in the US may cause economic or medical harm to the patient.^8,9^

Understanding that many formulary exclusions do not benefit patients^8,9^ and, as shown in this study, may affect large numbers of patients underlines the need for more transparency in how formularies are built and how rebate pricing affects these decisions. Biopharmaceutical costs for millions of patients comprise a large proportion of disproportionately high costs for disproportionately poor outcomes in the US compared with other developed countries. Similarly, the decision to include or exclude a medication on a formulary can affect outcomes and costs for large numbers of patients, not just a few using highly advanced medicines for rare indications. This study suggests that policies and legislation requiring transparent formulary decision-making and pricing are needed to fully understand whether and how formulary exclusions actually control costs or simply increase PBM and insurer profits.

It is also important to note that the calculated increases in untreated disease and adverse events carry costs that are often not factored into equations of medication cost-effectiveness. In addition, whether any of the substituted agents are truly more or less cost-effective cannot be determined in the current system in which the actual net prices of biopharmaceuticals are not transparent. What is certain, however, is that there are costs of forcing patients to switch to a nonequivalent medication that arise from untreated disease and increased adverse events. Those costs warrant further investigation.

### Limitations

Data on how many people use a particular medicine for a specific indication are limited, and the annual effects of medication switching can occur over many years. In some cases, we also had to utilize nonspecific and generalized prevalence data concerning discontinuation and switching. We modeled what would happen in the 2 largest formularies, but hundreds of unique formularies also exist, and each may have specific exclusions based on individual contracts negotiated. However, the data used to model the number of patients who could be affected are based on the most extensive available data sets. Thus, they can be considered an appropriate proxy for all formularies. Neither the prevalence of disease by severity and likelihood of adverse events or discontinuation nor the market share of a medication by age or other demographic characteristics are available. As a result, we can make no statements about how specific groups of patients could be affected. This study models a hypothetical situation based on known data and is not a hypothesis-testing statistical study to find correlations among variables.

## CONCLUSIONS

Evaluating the actual costs and benefits of formulary exclusions in the current US biopharmaceutical pricing environment is impossible when net prices paid by insurers and PBMs are not public. The unknown specific costs of forced switching further hamper the ability of economists and policymakers to perform cost-benefit analyses. We know that excluding medicines impacts hundreds of thousands of patients and increases morbidity and mortality. Forced medication substitutions due to formulary exclusions increases the burden of illness on patients and their care partners. It also increases costs for the ultimate payers in the healthcare system, patients, caregivers, employers, and the government. Determining these costs should be part of cost-benefit analyses of any formulary exclusion.

### Disclosures

R.P. is the founder of Conquest Advisors, LLC, owns stocks of biopharmaceutical companies, and was an employee of Pfizer for over two decades. He is a member of the Board of Councilors, University of Southern California, School of Pharmacy, Board of Directors, for University Pharmaco, LLC, Adjunct Clinical Faculty at Rutgers University, School of Pharmacy and serves as a consultant for the biopharmaceutical industry.

## Supplementary Material

Online Supplementary Material
