# Supplementary material for: Modeling the Effects of Formulary Exclusions: How Many Patients Could Be Affected by a Specific Exclusion?
Source: J Health Econ Outcomes Res. 2024 Mar 25;11(1):86–93. doi: 10.36469/001c.94544 (PMC10970716; doi:10.36469/001c.94544)
Supplement: Online Supplementary Material [file jheor_2024_11_1_94544_221311.pdf]

Journal of Health Economics and Outcomes Research

## Online Supplementary Material

Modeling the Effects of Formulary Exclusions: How Many Patients Could Be Affected by a Specific Exclusion. *JHEOR*. 2024;11(1):86-93. doi:10.36469/jheor.2024.94544

**Table S1: Data Sources Used for Calculation Shown in Figure 2**

This supplementary material has been provided by the authors to give readers additional information about their work.

This is an open-access article distributed under the terms of the Creative Commons Attribution 4.0 International License (CCBY-4.0). View this license's legal deed at http://creativecommons.org/licenses/by/4.0 and legal code at http://creativecommons.org/licenses/by/4.0/legalcode for more information.

**Table S1.** Data Sources Used for Calculation Shown in **Figure 2**

| | |
|---|---|
| Commercially insured lives | US Census Bureau[10] and Kaiser Family Foundation reports[11,12] |
| Indication prevalence | Results of literature review (see results section for specific references for each disease indication evaluated) |
| Formulary market share | *Drug Channels* article[13] |
| Medication market share | Data acquired from IQVIA |
| % likely to discontinue | Results of literature review (see results section for specific references for each disease indication evaluated) |
| % with adverse event | Results of literature review (see results section for specific references for each disease indication evaluated) |
